# Anti-Diabetic and Antioxidant Effect Evaluation of Thai Shallot and Cha-Miang in Diabetic Rats

**DOI:** 10.3390/biology14060627

**Published:** 2025-05-29

**Authors:** Jiraporn Laoung-on, Artorn Anuduang, Chalermpong Saenjum, Kittipan Rerkasem, Somdet Srichairatanakool, Kongsak Boonyapranai, Sakaewan Ounjaijean

**Affiliations:** 1 Office of Research Administration, Chiang Mai University, Chiang Mai 50200, Thailand; jiraporn.l@cmu.ac.th; 2 Research Institute for Health Sciences, Chiang Mai University, Chiang Mai 50200, Thailand; a.anuduang@gmail.com (A.A.); kongsak.b@cmu.ac.th (K.B.); 3 Department of Pharmaceutical Sciences, Faculty of Pharmacy, Chiang Mai University, Chiang Mai 50200, Thailand; chalermpong.saenjum@gmail.com; 4 Environmental-Occupational Health Sciences and Non Communicable Diseases Center of Excellence, Research Institute for Health Sciences, Chiang Mai University, Chiang Mai 50200, Thailand; rerkase@gmail.com; 5 Department of Biochemistry, Faculty of Medicine, Chiang Mai University, Chiang Mai 50200, Thailand; ssrichai@mail.med.cmu.ac.th

**Keywords:** hyperglycemia, type 2 diabetes mellitus, insulin resistance, metabolic syndrome, dyslipidemia, oxidative stress

## Abstract

Background: Type 2 diabetes mellitus is a chronic metabolic disease related to obesity and high-calorie diets, which presents hyperglycemia and insulin resistance. The management of diabetes can cause a rise in financial burden and synthetic medications can have adverse effects. This study aims to investigate the anti-diabetic and antioxidant activity of SHE, CME, and SHE in diabetic rats. Methods: Seventy-eight rats were randomly assigned to the following 13 groups (n = 6): N-DW, D-DW, D-M, D-S, D-C, D-F, D-SC, D-SF, D-SM, D-CM, D-FM, D-SCM, and D-SFM, for 8 weeks. Body weight, relative organ weight, blood glucose, insulin resistance, and plasma biomarkers related to diabetes were determined. Results: Diabetic rats had a significantly decreased body weight, RBC-GHS, liver-GHS, and RBC-SOD activity and a significantly increased relative organ weight, blood glucose levels, insulin levels, insulin resistance, HbA1C, TC, TG, LDL, plasma-MDA, and liver-MDA when compared to the N-DW group. However, plant supplementation and metformin improved diabetes-related parameters, especially the combination of CME or FCME with SHE and metformin, which showed the greatest potential for improving diabetic conditions. (4) Conclusions: The research indicates that CME or FCME conjugated with SHE and metformin demonstrates the greatest potential for enhancing diabetic conditions, confirming positive synergism. These findings suggest that the combination of CME or FCME with SHE and metformin may be effective for diabetic treatment.

## Simple Summary

Our research focused on plant supplementation, including SHE, CME, and FCME alone, combinations of plants, and combinations of plants and synthetic drugs, which influence the best potential to treat diabetes-related conditions. Diabetic rats were treated with a single plant, combinations of plants, and combinations of plants with metformin. Our data showed that the combination of CME or FCME with SHE and metformin showed the greatest potential to improve diabetes-related conditions, indicating positive synergism. These results reinforce the evidence that a combination of synthetic medications and medicinal plants can significantly impact diabetes treatment.

## 1. Introduction

Diabetes mellitus (DM) is a chronic metabolic disorder characterized by hyperglycemia, insulin resistance, and abnormalities in carbohydrate, lipid, and protein metabolism [1]. This condition has considerable systemic implications, especially type 2 DM, which results in chronic hyperglycemia due to insulin resistance and reduced pancreatic β-cell production [2]. Type 2 DM progressively affects several organ systems, leading to negative repercussions, including cardiovascular disease, neuropathy, renal failure, and infections [3]. Lifestyle choices, such as high-calorie diets, sedentary behavior, and obesity, are significantly associated with the onset and development of type 2 DM [4].

In Thailand, the prevalence of type 2 DM is increasing due to unhealthy diets, rising obesity rates, and an aging population. The incidence rate of type 2 DM has shown an increasing trend over time, ranking fifth among males and third among females in the top ten diseases [5]. The management of diabetic patients and their associated complications incurs significant costs, leading to a rise in financial burden for the government [5]. People with diabetes use insulin and synthetic anti-diabetic medications to regulate their glucose levels. Progressively, these medications may exhibit a reduced efficacy and can lead to adverse effects such as nausea, vomiting, and diarrhea [6]. The prolonged administration of insulin may lead to insulin resistance, cerebral atrophy, anorexia, and hepatic steatosis [7]. Natural herbs or plants exhibiting anti-diabetic properties have been utilized for the prevention and management of diabetes [7]. Medicinal plants offer a reliable, cost-effective, and secure option for health and wellness. Moreover, medicinal plants utilized alongside synthetic medications may enhance drug efficacy, decrease drug dosage, minimize adverse effects, and assist in normalizing blood glucose levels [8]. However, the integration of natural and synthetic treatments has the potential to enhance or minimize their effectiveness, which depends on the mechanisms at play and their interaction. Therefore, it is essential to conduct research to identify beneficial plants for diabetes management and to compare their effects with synthetic medications prior to patient treatment.

Southeast Asian shallots (*Allium ascalonicum* L.) are a variety of red onion. This plant has been used in Asian cuisines and traditional medicine for managing fevers, flatulence, and infections. Shallots are abundant in allin, allicin, flavonoids, and phenolic compounds [9]. These compounds are powerful natural antioxidants exhibiting antibacterial, antiviral, anti-diabetic, and anti-inflammatory properties [10]. Consequently, shallot extract was examined for its efficacy in mitigating free radical damage and treating oxidative disorders [9]. Tea (*Camellia sinensis*) is cultivated and consumed globally, and cha-miang (*Camellia sinensis* var. *assamica*) is the predominant variety cultivated in northern Thailand [11]. Its biological features include properties that fight against oxidation, mutations, and cancer; the prevention of certain chemical reactions; and a reduction in tumors due to phenolic compounds [11]. Gallic acid, epigallocatechin, caffeine, catechin, epicatechin, and gallocatechin gallate have demonstrated the ability to inhibit cancer, hypertension, inflammation, diabetes, obesity, hyperlipidemia, and cardiovascular disease [12,13].

Despite the popularity of shallot and tea as medicinal plants, the combination of Thai shallot, cha-miang, and synthetic medicine (metformin) for anti-diabetic and antioxidant applications has not been studied. Therefore, this study aims to investigate the anti-diabetic and antioxidant activity of this combination and its effect on streptozotocin (STZ)-induced diabetic rats.

## 2. Materials and Methods

### 2.1. Shallot and Cha-Miang Extracts

Fresh Thai shallot bulbs (*Allium ascalonicum* cv. *Chiangmai*) from northern Thailand (Sanpatong District, Chiang Mai, Thailand) were harvested from March to April 2017. The Herbarium Curator at The Botanical Garden Organization, Queen Sirikit Botanic Garden, Mae Rim, Chiang Mai, deposited and authenticated the plant materials (SK-SH-CM01). The shallot extract (SHE) was produced according to previous studies which showed its high phenolic and flavonoid contents [9]. HPLC analysis of the SHE showed the presence of total quercetin (9.07 ± 0.11 mg/g plant extract), including 52% of quercetin aglycone and 48% of quercetin glycosides.

Cha-miang (*Camellia sinensis* var. *assamica*) from northern Thailand (Chiang Dao District, Chiang Mai, Thailand) was harvested in September 2016. Specimens were deposited at the Research Institute for Health Sciences, Chiang Mai University (00159). Fresh cha-miang leaf extract (CME) and fermented cha-miang leaf extract (FCME) were prepared according to a previous study [14] which showed their high phenolic and flavonoid contents. HPLC analysis of the CME and FCME showed the presence of catechin and related compounds [15].

The SHE, CME, and FCME were stored at −20 °C before experimentation.

### 2.2. Experimental Animals

Seventy-eight (8 weeks old) male Wistar rats were purchased from the animal house of the National Laboratory Animal Center at Mahidol University (Nakhon Pathom, Thailand). The animals were housed by three in each cage with a 25 ± 2 °C temperature and 12 h light/dark cycle. They were acclimatized before the experimental procedures for two weeks. Rats were sustained on a commercially balanced diet and water. All experimental protocols received approval from the Animal Ethics Committee, Faculty of Medicine, Chiang Mai University (38/2559) and followed with institutional guidelines for the Care and Use of Laboratory Animals.

### 2.3. Experimental Design

Seventy-eight male rats were randomly assigned to 13 groups (*n* = 6 per group). Group I was fed a normal control diet (NCD), whereas male rats in Groups II–XIII were induced to be insulin-resistant using a high-fat diet (HFD) for 4 weeks. After acclimatization and induction by HFD, a single dose of 35 mg/kg of streptozotocin (STZ) in citrate buffer, pH 4.6, was injected intraperitoneally for diabetic induction. Diabetes was confirmed with a blood sample collected from the tail tip, with a fasting blood glucose (FBG) level exceeding 200 mg/dL being considered as diabetic. Then, the treatments were assigned as follows:

- Group I (control: N-DW): rats in this group were orally administered 1 mL/day of distilled water.
- Group II (Diabetic control: D-DW): rats were orally administered 1 mL/day of distilled water.
- Group III (Positive control: D-M): rats were orally administered metformin (Met) at doses 100 mg/kg BW.
- Group IV (Diabetic with SHE: D-S): rats in this group were orally administered SHE 1000 mg/kg BW.
- Group V (Diabetic with CME: D-C): rats in this group were orally administered CME 300 mg/kg BW.
- Group VI (Diabetic with FCME: D-F): rats in this group were orally administered FCME 300 mg/kg BW.
- Group VII (Diabetic with SHE + CME: D-SC): rats in this group were orally administered SHE and CME 300 mg/kg BW.
- Group VIII (Diabetic with SHE + FCME: D-SF): rats in this group were orally administered SHE 1000 mg/kg BW and FCME 300 mg/kg BW.
- Group IX (Diabetic with SHE + Met: D-SM): rats in this group were orally administered SHE 1000 mg/kg BW and Met 100 mg/kg BW.
- Group X (Diabetic with CME + Met: D-CM): rats in this group were orally administered CME 300 mg/kg BW and Met 100 mg/kg BW.
- Group XI (Diabetic with FCME + Met: D-FM): rats in this group were orally administered FCME 300 mg/kg BW and Met 100 mg/kg BW.
- Group XII (Diabetic with SHE + CME + Met: D-SCM): rats in this group were orally administered SHE 1000 mg/kg BW, CME 300 mg/kg BW, and Met 100 mg/kg BW.
- Group XIII (Diabetic with SHE + FCME + Met: D-SFM): rats in this group were orally administered SHE 1000 mg/kg BW, FCME 300 mg/kg BW, and Met 100 mg/kg BW.

The recommended dose of SHE is 1000 mg/kg, which corresponds with the safe and effective dose established in a subacute toxicity study (non-published data). Moreover, the CME and FCME in the current research were administered according to the safe and effective dose established in a subacute toxicity study, which was 300 mg/kg [15]. All groups received daily oral treatment via gavage for a duration of 8 weeks.

### 2.4. Measurement of Body Weight and Organ Weight

Body weight and food consumption were recorded weekly. At the end of the experimental period, the animals were fasted overnight and anesthetized by an intraperitoneal injection of pentobarbital sodium salt (150 mg/kg BW). Blood was collected via hepatic portal vein for biochemical parameter analysis. The livers, kidneys, spleen, heart, and pancreas were removed, prepared, and weighed.

### 2.5. Oral Glucose Tolerance Test (OGTT)

At the end of the experimental period, rats underwent 4 h of extract deprivation prior to oral gavage with a glucose solution (2 g/kg BW). Blood samples were collected at 0, 15, 30, 45, 60, 90, and 120 min after glucose administration. Glycaemia was assessed with a glucose meter (Accu Chek Performa, Roche, IN, USA). We determined blood glucose levels at various intervals, shown as a percentage of the alteration in blood glucose from the baseline measurement.

### 2.6. Determination of Fasting Blood Glucose, Plasma Insulin, and Glycated Hemoglobin

Fasting blood glucose was assessed using a glucose meter (Accu Chek Performa, Roche, IN, USA) after overnight fasting. Serum insulin levels were measured using the Rat Ins1/Insulin ELISA Kit (Sigma, Burlington, NJ, USA), and the homeostasis model assessment of insulin resistance (HOMA-IR) was calculated using the following equation [16]:

HOMA-IR = [fasting insulin (µU/mL) × fasting glucose (mmol/L)]/22.5

The glycated hemoglobin levels were measured using the HbA1C assay kit (Randox-HA3830) (Randox Laboratories Ltd., Antrim, UK).

### 2.7. Biochemical Analysis

Blood samples were collected biweekly via tail vein sampling to assess biochemical indicators of obesity and hyperlipidemia. Plasma cholesterol (Randox-RA-CH3810S2), plasma triglyceride (Randox-RA-TR3823S1), LDL-cholesterol levels(Randox-RA-CH3841S3), alanine aminotransferase (ALT) (Randox-RA-AL3801S1), aspartate aminotransferase (AST) (Randox-RA-AS3804S1), blood urea nitrogen (BUN) (Randox-UR3825S1), and creatinine (Randox-CR3814S3) were quantified utilizing an automatic analyzer (Randox, West Virginia, CA, USA). All procedures were followed according to the manufacturer’s guidelines.

### 2.8. Antioxidant Assessment

Lipid peroxidation (LPO) in plasma and hepatic tissue was assessed with the thiobarbituric-acid-reactive substance (TBAR) method. The antioxidant level was determined using the superoxide dismutase (SOD) enzyme in plasma, utilizing an automatic analyzer (Randox, West Virginia, CA, USA). In addition, glutathione peroxidase (GPx) and glutathione (GSH) were measured using commercial ELISA kits (ab102530 and CS0260, respectively) from Abcam (Waltham, MA, USA) and Sigma-Aldrich (Burlington, NJ, USA).

### 2.9. Histological Evaluation

The liver, kidneys, spleen, pancreas, and heart of each animal were fixed in a 10% formaldehyde solution for paraffin section, with a section thickness of 3 µm. The slide was stained with hematoxylin and eosin (H&E) and the morphological features of each organ were examined using a light microscope (Olympus CX31, Olympus Corporation, Tokyo, Japan) at 100×.

### 2.10. Statistical Analysis

The data were distributed as descriptive statistics, expressed as mean ± standard error of the mean (SEM). The Kolmogorov–Smirnov test was used to evaluate normal distributions. The mean values of all parameters were obtained using one-way ANOVA, followed by Duncan’s multiple-range test to analyze the differences between groups. All statistical analyses were conducted with SPSS 22 statistical software (Chicago, IL, USA). Statistical significance was accepted at *p* < 0.05.

## 3. Results

### 3.1. Effect of Plant Supplementation Consumption on Body Weight and Relative Organ Weight

The rats receiving the high-fat diet experienced a significant body weight increase when compared to rats receiving the normal diet. After the injection of streptozotocin to induce diabetes, the diabetic rats showed a significant decrease in weight, which gradually recovered when given a continuous high-fat diet. However, their weight remained substantially lower when compared to the control rats throughout the duration of the experiment.

After treatment with the plant supplementations and metformin for 8 weeks, all treatments caused a significantly increased weight when compared to the D-DW group, which was similar to the N-DW group, except the rats treated with metformin (Table 1).

The D-DW group had significantly increased relative liver, kidney, and heart weights when compared to the control. The D-SF, D-SM, and D-FM groups had a significantly decreased relative liver weight compared to the D-DW group, but it was still higher than that of the N-DW group. Moreover, the D-SC, D-SCM, and D-SFM groups had a significantly decreased relative liver weight when compared with the D-DW group, the same as the N-DW group. Additionally, all treatment groups had significantly decreased relative kidney and heart weights when compared to the D-DW group. However, there were no significant differences in the relative weights of the spleen, pancreas, and white adipose tissue (WAT) from epididymal when compared to the N-DW and D-DW groups (Table 1).

### 3.2. Effect of Plant Supplementation Consumption on OGTT

**Figure 1:**
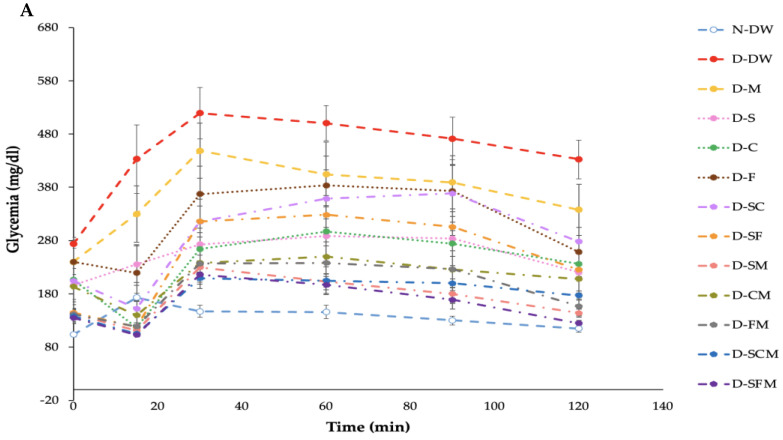

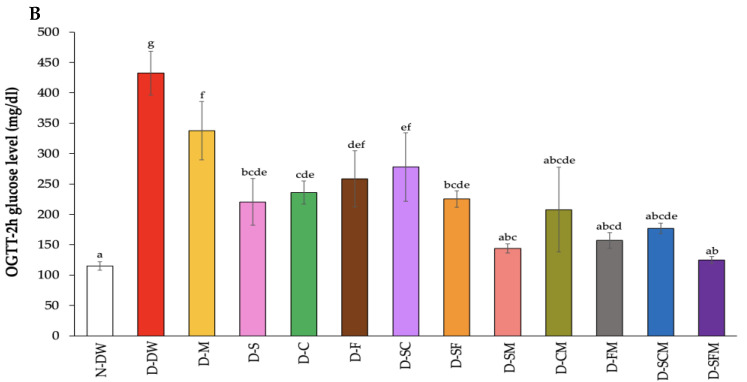
Effect of SHE, CME, and FCME on OGTT. Plasma glucose concentrations were measured throughout a 0–120 min interval during the OGTT (**A**), and glucose levels at 120 min were recorded and analyzed (**B**). Values were analyzed by the One-Way ANOVA followed by the Duncan’s test (n = 6). ^a,b,c,d,e,f,g^ Denotes the different letters indicate significant differences between groups at *p* ≤ 0.05. The data are demonstrated as the mean ± SEM (error bar).

The N-DW, D-DW, D-M, and D-S groups demonstrated increased glycemia within 15 min after oral gavage of 2 g/kg BW of glucose, while other groups showed an increase in 30 min. The D-DW group showed the highest significant glycemic response at all time intervals, reaching its maximum at 30 min, thereafter declining while remaining higher compared to other groups. In contrast, the N-DW group presented a minimal glycemic response throughout the whole study duration. The different plant supplement consumptions, pharmacological treatments, and various combinations showed moderate glycemic levels, indicating varying degrees of glycemic control. Most groups exhibited the highest glycemia at 30 min post-treatment, followed by a general decline toward baseline values by 120 min (Figure 1A).

The mean blood glucose levels of the D-DW group presented a significant increase when compared to the N-DW group. All treatment groups had significantly reduced blood glucose levels when compared with the D-DW group. The combination treatment, especially in the D-CM, D-FM, D-SCM, and D-SFM groups, had significantly reduced glycemic values when compared to the D-DW and D-M groups, which were similar to the N-DW group (Figure 1B).

### 3.3. Effect of Plant Supplementation Consumption on Fasting Blood Glucose, Plasma Insulin, and Glycated Hemoglobin

**Figure 2:**
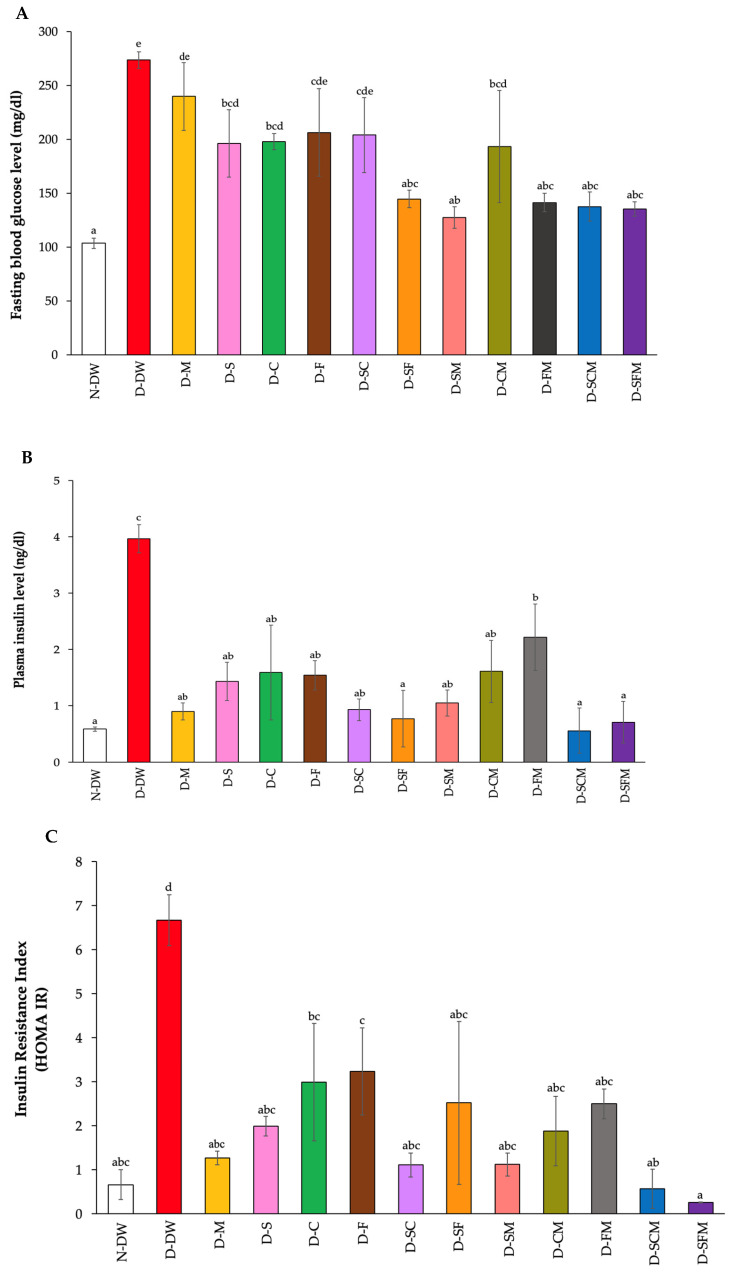

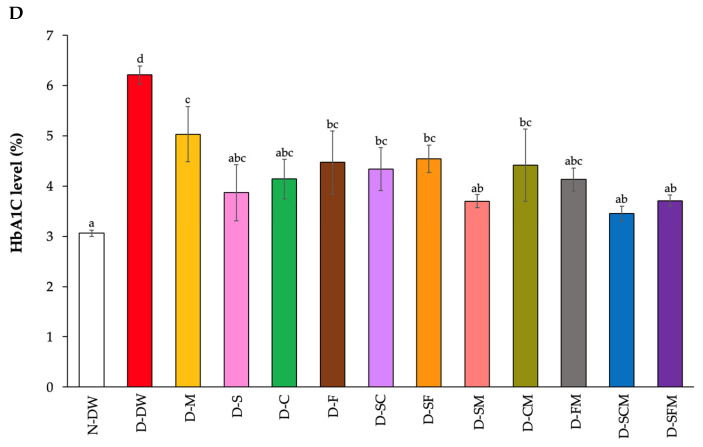
Effect of SHE, CME, and FCME on fasting blood glucose, plasma insulin, and glycated hemoglobin. Fasting blood glucose levels were measured (**A**). The insulin levels (**B**), insulin resistance index (**C**), and the proportion of HbA1C (**D**) were measured and analyzed. Values were analyzed by the One-Way ANOVA followed by the Duncan’s test (*n* = 6). ^a,b,c,d,e^ Denotes the different letters indicate significant differences between groups at *p* ≤ 0.05. The data are demonstrated as the mean ± SEM (error bar).

The D-DW group had a significantly increased fasting blood glucose level when compared to the N-DW group. However, all treatment groups had significantly decreased fasting blood glucose levels when compared with the D-DW rats, especially the D-SF, D-SM, D-FM, D-SCM, and D-SFM groups, which presented blood glucose levels the same as the N-DW group (Figure 2A). Additionally, the plasma insulin level of the D-DW group demonstrated a significant increase when compared to the N-DW group. All treatment groups had significantly decreased fasting blood glucose levels when compared with D-DW group, the same as the N-DW group, except for the D-CM group (Figure 2B). Moreover, the D-DW rats showed a significant increase in insulin resistance index (HOMA IR) compared to the N-DW group, whereas all treatments of diabetic rats significantly reduced the insulin resistance index to levels that were similar to the N-DW group (Figure 2C). Furthermore, the glycohemoglobin (HbA1C) of the D-DW group showed a significant increase when compared with the N-DW rats. All treatment groups saw a significantly decline in the proportion of HbA1C when compared to the D-DW rats. The D-SF, D-SM, D-FM, D-SCM, and D-SFM groups significantly decreased in the proportion of HbA1C when compared to the D-M rats, which were similar to the N-DW group (Figure 2D).

### 3.4. Effect of Plant Supplementation Consumption on Biochemical Parameters

The plasma biochemical parameters, which include AST, ALT, BUN, creatinine, total cholesterol (TC), total triglyceride (TG), and LDL-cholesterol, are displayed in Table 2. The D-DW group had significantly increased AST, ALT, BUN, TC, TG, and LDL levels when compared to the N-DW group. All treatment groups had significantly declined AST, ALT, BUN, and TG levels compared to the D-DW group, similar to the N-DW group, except for the BUN level of the D-SCM group. Moreover, the total cholesterol level significantly decreased in all groups, although it was higher than in the N-DW and D-M groups. Additionally, all treatment groups, except the D-C, D-SM, D-CM, and D-SCM groups, showed significantly decreased LDL-cholesterol levels compared to the D-DM group, which was similar to the N-DW group. However, there were no significant differences in the creatinine level when compared to the N-DW and D-DW groups.

### 3.5. Effect of Plant Supplementation Consumption on Antioxidant Activity

The levels of plasma MDA and liver MDA were significantly increased in the D-DW group when compared to the N-DW group. The plasma MDA level was significantly decreased in all treatment groups when compared with the D-DW and D-M groups. Furthermore, all treatment groups, except for the D-S, D-C, and D-SC groups, experienced a significant decline in liver MDA levels when compared to the D-DW group. The antioxidant status was changed in RBC-GHS, liver-GHS, and RBC-SOD. The D-DW rats significantly declined in RBC-GHS, liver-GHS, and RCB-SOD when compared to the N-DW groups, whereas all treatment groups, except the D-S and D-SC groups, had a significantly improved RBC-GHS compared to the D-DW rats. The D-DW group saw a significant drop in RBC-SOD compared to the N-DW rats, but all treatments improved RBC-SOD levels except for in the D-SF, D-FM, and D-SFM groups. However, the RBC-GPx of the D-DW rats was not significantly different from the N-DW group, while the D-SM, D-FM, and D-SFM groups had significantly higher RBC-GPx levels than the D-DW group (Table 3).

### 3.6. Effect of Plant Supplementation Consumption on Organs Histological Features

**Figure 3:**
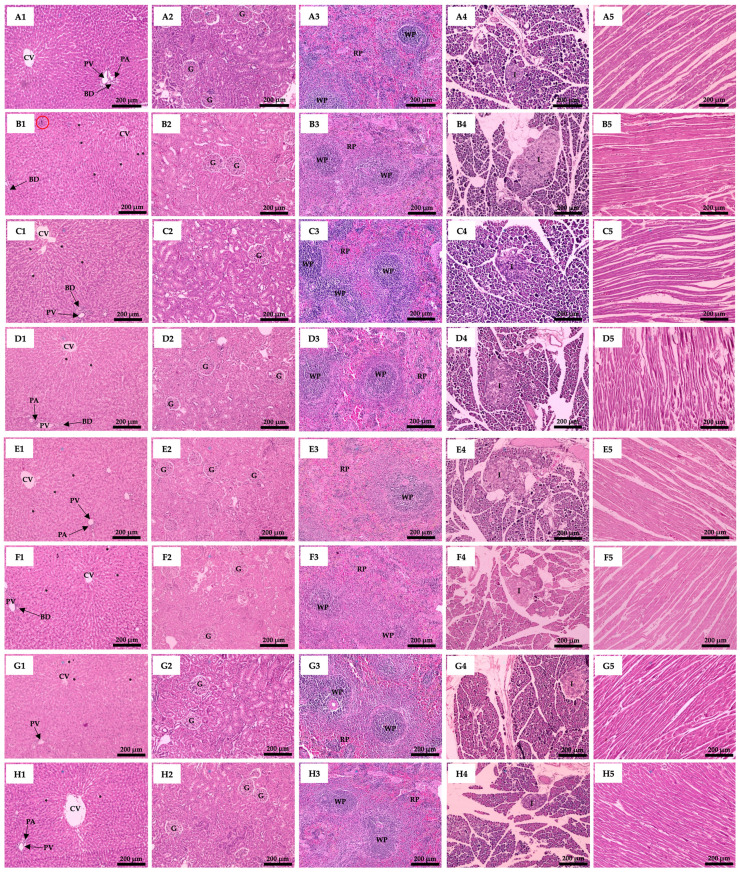

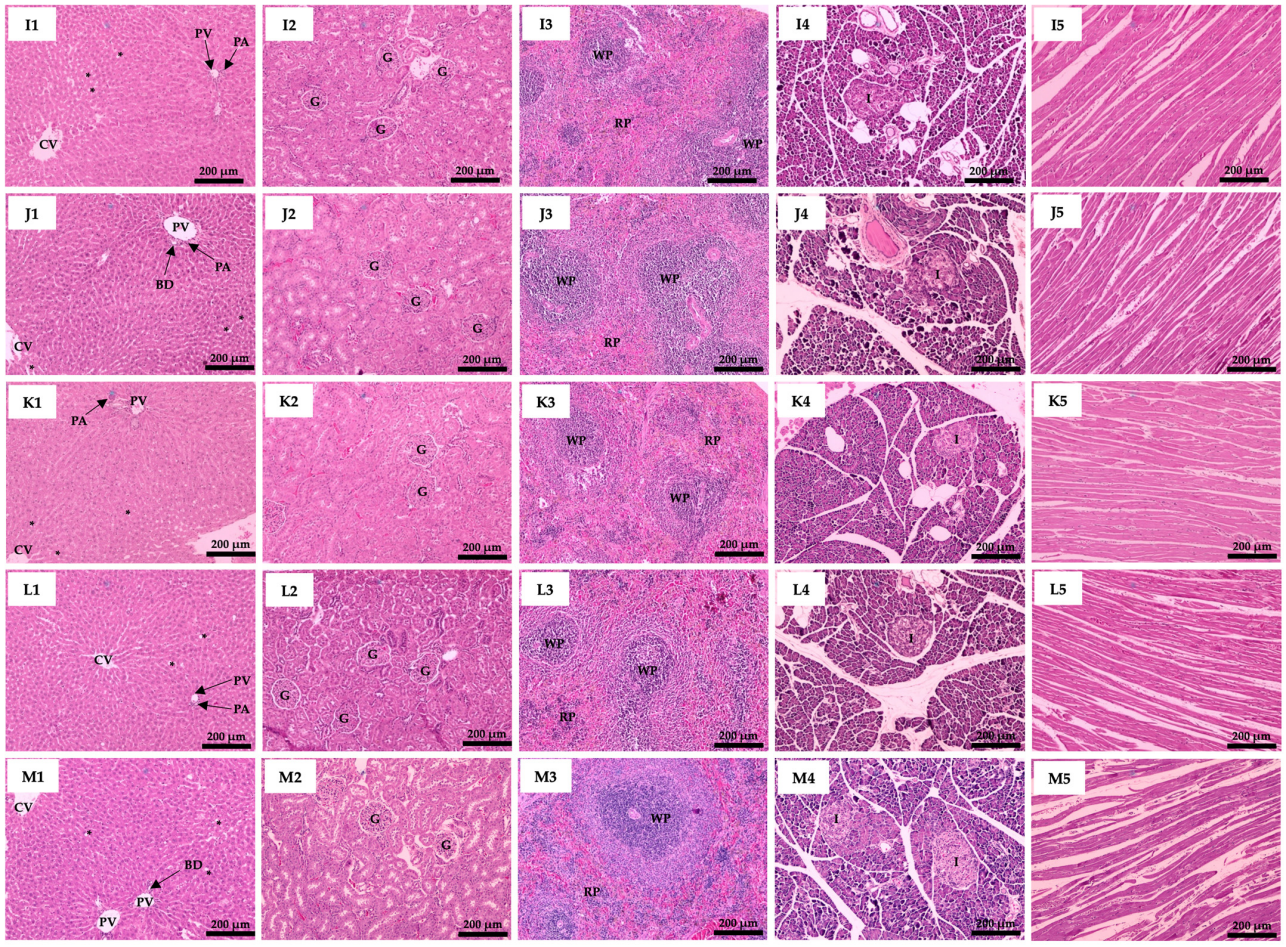
Photomicrographs of rat liver, kidney, spleen, pancreas, and heart using H&E staining and showed at the magnification of 100×. (**A1–A5**) N-DW; (**B1–B5**) D-DW; (**C1–C5**) D-M; (**D1–D5**) D-S; (**E1–E5**) D-C; (**F1–F5**) D-F; (**G1–G5**) D-SC; (**H1–H5**) D-SF; (**I1–I5**) D-SM; (**J1–J5**) D-CM; (**K1–K5**) D-FM; (**L1–L5**) D-SCM; and (**M1–M5**) D-SFM. CV: central vein; PV: portal vein; PA: portal artery; BD: bile duct; *: lipid droplets; red circle: Kupffer cells; G: glomerulus; WP: white pulp; RP: red pulp; I: Islet of Langerhans.

The histological features of the liver, kidney, spleen, pancreas, and heart are illustrated in Figure 3. The liver tissues showed that the diabetic rats saw increased fat accumulation in the liver (fatty liver). Lipid droplets of different sizes were found, indicating the progression of non-alcoholic fatty liver disease (NAFLD). Moreover, in the liver tissue of D-DW rats, groups of Kupffer cells were found, which indicated that oxidative stress and inflammation were occurring. The hepatic histological analysis of other groups did not demonstrate a group of Kupffer cells (Figure 3). However, the histological characteristics of other organs stained with H&E showed no differences among all groups.

## 4. Discussion

In this study, rats were induced into metabolic abnormality by a high-fat diet (HFD) and an experimental diabetic model induced by streptozotocin (STZ) was produced. Diabetes mellitus (DM) is a chronic metabolic disorder resulting in hyperglycemia, increased total cholesterol (TC), triglycerides (TG), and low-density lipoprotein (LDL) cholesterol [17]. This study found that the rats receiving the HFD experienced a continuous increase in body weight compared to the normal control over 4-week period before the induction of diabetes with STZ, which indicates that the HFD produced obesity through dysregulated energy [18]. However, diabetic rats (D-DW) demonstrated a significant body weight reduction after STZ injection, despite continuous high-fat diet feeding, and demonstrated increased relative liver, kidney, and heart weights when compared to non-diabetic controls (N-DW). Similarly, a single dose of STZ-induced diabetes caused a significant reduction in body weight, whereas the relative liver and kidney weights were increased [19]. A reduction in body weight and organ atrophy reflect the metabolic disfunction and systematic inflammation associated with diabetes progression [20]. Diabetes mellitus (DM) is a metabolic disorder in which body tissues lose their capacity to use glucose, resulting in increased protein utilization and consequent weight reduction [21]. The diabetic rats treated with plant supplements saw significantly improved body weight recovery when compared to the D-DW rats, perhaps resulting from enhanced glycemic regulation and the improved production of protein structures [22]. Moreover, relative liver, kidney, and heart weights were significantly reduced in the diabetic rats treated with plant supplements, especially with combination treatments, including the D-SC, D-SCM, and D-SFM groups, which showed similar results to the N-DW group. This may have been achieved through a reduction in hepatic glycogenesis and glucose release from the liver, with the suppression of lipolysis in adipose tissue and organ inflammation [23]. The potential mechanism for the enhancement of body weight and the reduction in relative essential organ weight in rats treated with plant supplements may be attributed to extra pancreatic actions that could have facilitated greater glucose utilization by tissues and organs [19].

The oral glucose tolerance test (OGTT) indicated that diabetic rats exhibited impaired glucose clearance, with sustained elevated blood glucose levels during the testing period. All treatments significantly enhanced glycemic profiles, especially the combination treatments, including in the D-CM, D-FM, D-SCM, and D-SFM groups, which demonstrated the most effective glycemic control, nearly at the levels of healthy controls. Similarly, previous reports demonstrated that *Catharanthus roseus* and *Cocconia cordifolia* had the potential to reduce blood glucose levels at 30 and 60 min after oral glucose consumption, respectively [24]. Additionally, the decoction of a combination of *Vernonia amygdalina*, *Gongronema latifolium*, and *Occimum gratissimum* was found to be superior in the activity of one plant extract [25]. These results indicate that plant combinations with metformin may enhance insulin sensitivity and facilitate more effective glucose application [24], potentially exceeding the effects of metformin alone, confirming positive synergism.

The results for fasting blood glucose (FBG), plasma insulin, insulin resistance (HOMA-IR), and glycated hemoglobin (HbA1C) were consistent with the results of the OGTT assessment. The D-DW rats had significantly increased FBG, plasma insulin, HOMA-IR, and HbA1C when compared to the N-DW rats. Diabetes is a chronic metabolic disorder characterized by the impaired metabolism of carbohydrates, proteins, and lipids, leading to hyperglycemia [26]. Hyperglycemia, a defining feature of type 2 DM, may contribute to severe complications because of its progressive and ongoing nature [27]. In addition, rising fasting plasma insulin levels are predictive of type 2 diabetes, a condition referred to as hyperinsulinemia [28]. The diabetic rats receiving all treatments showed reduced FBG, plasma insulin levels, HOMA-IR, and HbA1C compared to the diabetic rats, especially with the combination of plant extracts and metformin. Corresponding to previous reports, medicinal plants with hypoglycemic activity depend on various mechanisms, including the enhancement of insulin sensitivity in target cells, the stimulation of insulin secretion, and the promotion of β-cell regeneration in the islets of Langerhans in the pancreas [29]. A previous report found that flavonoids and phenolics are strong natural antioxidants, which contribute to antidiabetic activity [30]. Furthermore, flavonoids have been recognized for their ability to regenerate damaged β-cells in diabetic rats and function as secretions of insulin [31]. The SHE, CME, and FCME were rich in phenolics and flavonoids, which significantly reduced blood glucose levels. This reduction may be attributed to the regeneration of pancreatic cells, which, in turn, leads to improved insulin secretion.

The diabetic rats demonstrated increased levels of AST, ALT, and BUN, which are enzymes of the liver and kidney and are considered as good biomarkers of hepatotoxicity and nephrotoxicity [32]. In D-DW rats, increased levels of these enzymes were possibly due to hepatic cell and kidney cell damage [32,33]. The diabetic rats that received plant supplements had lower levels of AST, ALT, and BUN, almost back to normal, which shows that the supplements helped to protect the liver and kidneys by keeping their cells healthy and improving liver function in diabetic rats [22]. The diabetes control group had higher levels of TC, TG, and LDL-cholesterol, while all treatments lowered these levels, except for in the D-C, D-SM, D-CM, and D-SCM groups, which showed no change in LDL-cholesterol compared to the diabetic rats. Diabetes frequently results in hyperglycemia and hyperlipidemia, where cholesterol, triglycerides, and LDL-cholesterol serve as the primary risk factors for cardiovascular diseases [34]. In diabetes, the liver converts excess serum fatty acids into phospholipids, cholesterol, and triglycerides, which then enter the bloodstream as lipoproteins [35]. The decline in cholesterol, triglycerides, and LDL-cholesterol levels suggests that SHE, CME, and FCME may reduce the risk of cardiovascular conditions.

Additionally, the D-DW group had increased plasma-MDA and liver-MDA and decreased RBC-GHS, liver-GHS, and RBC-SOD activity when compared to the N-NW group. However, the SHE-, CME-, and FCME-supplemented diabetic rats had enhanced RBC-SOD and reduced plasma-MDA and liver-MDA compared to the D-DW group. Similarly, the previous study found that CME and FCME had the potential to improve SOD activity and decrease plasma and liver MDA in obese rats [36]. In addition, the shallot extract at low doses had enhanced SOD production in the endothelial cell line [9]. Oxidative stress is the one factor that contributes to DM, which occurs when continuously high blood glucose levels induce reactive oxygen species (ROS), resulting in tissue damage [37]. Several studies have found that phytochemicals such as tannins, kaempferol, flavonoids, and phenolics present anti-inflammatory [9], antioxidant [9,38], neuroprotective [39], and hepatoprotective activity [1,36,40].

All rats receiving high-fat diets presented increasing lipid droplets in liver histology when compared to the normal diet rats. A high-calorie diet causes obesity, which increases adipocyte size by altering the adipose tissue structure and cellular composition [41]. Normal hepatic lipid droplets may expand due to lipid transport and lipoprotein secretion changes [42]. Moreover, the liver histological evaluation of D-DW rats found groups of Kupffer cells, which indicated that hepatocyte damage and lipid peroxidation induced an inflammatory response. This has been correlated with STZ-induced diabetic mellitus that causes liver damage [43,44]. The group of Kupffer cells was not found in all treatment groups, which indicated that SHE, CME, FCME, and metformin were beneficial in regulating liver lipid peroxidation and the inflammatory response.

The present study demonstrates that SHE, CME, FCME, and metformin, especially when used in combination with metformin, SHE, and CME or FCME, showed a more advantageous impact on numerous diabetes-related parameters. The CME or FCME combination with SHE and metformin showed anti-diabetic effects by regulating insulin secretion and inhibiting hyperlipidemia and hyperglycemia, hence diminishing damage-related liver and kidney enzymes. As a result, the combination of CME or FCME with SHE and metformin was found to be very helpful for diabetes, and our research provides a solid basis for using it to treat this condition.

## 5. Conclusions

In conclusion, our findings indicate that SHE, CME, and FCME reduce diabetes and its related complications in rats. SHE, CME, and FCME are associated with an increase in body weight and a decrease in relative liver, kidney, and heart weights compared to the D-DW group. SHE, CME, and FCME reduce blood glucose levels, plasma insulin levels, insulin resistance, and plasma biomarkers related to diabetes. The combination of CME or FCME with SHE and metformin demonstrates the greatest potential for enhancing diabetic conditions, thereby confirming positive synergism. These findings suggest that the combination of CME or FCME with SHE and metformin may be an effective option for diabetic treatment.

## Figures and Tables

**Table 1:** Effect of SHE, CME, and FCME on body weight and organ weight.

Groups	Body Weight (g)	Relative Organs Weight (g/100 g BW)					
		Livers	Kidneys	Spleen	Heart	Pancreas	WAT
N-DW	554.00 ± 8.72 ^bc^	2.00 ± 0.02 ^a^	0.19 ± 0.01 ^a^	0.16 ± 0.01	0.26 ± 0.01 ^ab^	0.24 ± 0.02	6.12 ± 0.24
D-DW	446.25 ± 21.35 ^a^	3.02 ± 0.17 ^f^	0.27 ± 0.02 ^c^	0.19 ± 0.02	0.34 ± 0.02 ^c^	0.31 ± 0.03	7.91 ± 0.69
D-M	497.50 ± 31.46 ^ab^	2.79 ± 0.15 ^ef^	0.23 ± 0.01 ^b^	0.16 ± 0.01	0.28 ± 0.02 ^b^	0.30 ± 0.02	7.68 ± 0.69
D-S	547.50 ± 12.50 ^bc^	2.69 ± 0.10 ^def^	0.22 ± 0.02 ^ab^	0.17 ± 0.01	0.26 ± 0.02 ^ab^	0.30 ± 0.01	7.33 ± 0.92
D-C	557.50 ± 11.09 ^bc^	2.63 ± 0.12 ^cdef^	0.20 ± 0.01 ^ab^	0.15 ± 0.01	0.24 ± 0.01 ^ab^	0.26 ± 0.01	8.28 ± 1.00
D-F	530.00 ± 17.32 ^bc^	2.63 ± 0.03 ^cdef^	0.22 ± 0.01 ^ab^	0.15 ± 0.01	0.27 ± 0.01 ^ab^	0.26 ± 0.02	8.39 ± 0.80
D-SC	522.50 ± 32.24 ^b^	2.22 ± 0.26 ^abc^	0.22 ± 0.01 ^ab^	0.16 ± 0.01	0.27 ± 0.01 ^ab^	0.29 ± 0.02	8.29 ± 1.25
D-SF	591.25 ± 29.18 ^c^	2.49 ± 0.24 ^bcde^	0.20 ± 0.01 ^ab^	0.15 ± 0.02	0.24 ± 0.02 ^ab^	0.28 ± 0.03	10.37 ± 1.29
D-SM	537.50 ± 10.31 ^bc^	2.46 ± 0.07 ^bcde^	0.22 ± 0.01 ^ab^	0.18 ± 0.01	0.26 ± 0.01 ^ab^	0.28 ± 0.03	7.39 ± 0.39
D-CM	551.25 ± 22.95 ^bc^	2.80 ± 0.18 ^ef^	0.23 ± 0.01 ^b^	0.17 ± 0.01	0.25 ± 0.01 ^ab^	0.31 ± 0.02	7.87 ± 0.30
D-FM	537.00 ± 15.46 ^bc^	2.52 ± 0.11 ^bcde^	0.21 ± 0.01 ^ab^	0.15 ± 0.01	0.24 ± 0.01 ^ab^	0.31 ± 0.03	7.89 ± 0.62
D-SCM	550.00 ± 17.80 ^bc^	2.32 ± 0.08 ^abcd^	0.21 ± 0.01 ^ab^	0.17 ± 0.01	0.26 ± 0.02 ^ab^	0.29 ± 0.02	8.58 ± 0.68
D-SFM	531.25 ± 9.66 ^bc^	2.18 ± 0.03 ^ab^	0.21 ± 0.01 ^ab^	0.18 ± 0.01	0.24 ± 0.01 ^a^	0.29 ± 0.01	8.26 ± 0.61

Body weight and relative organ weight were analyzed by One-Way ANOVA followed by the Duncan’s test (*n* = 6). ^a,b,c,d,e,f^ Denotes the different letters indicate significant differences between groups at *p* ≤ 0.05. The data are demonstrated as the mean ± standard error of mean (SEM).

**Table 2:** Effect of SHE, CME, and FCME on biochemical parameters.

Groups	AST (U/L)	ALT (U/L)	BUN (mg/dL)	Creatinine (mg/dL)	TC (mg/dL)	TG (mg/dL)	LDL (mg/dL)
N-DW	99.01 ± 7.81 ^a^	38.85 ± 3.04 ^a^	15.25 ± 0.81 ^ab^	1.12 ± 0.05	75.67 ± 2.51 ^a^	110.83 ± 8.42 ^abc^	26.26 ± 3.22 ^a^
D-DW	156.92 ± 14.69 ^b^	92.86 ± 1.32 ^b^	20.79 ± 2.64 ^c^	1.41 ± 0.30	144.00 ± 5.05 ^d^	248.50 ± 33.07 ^d^	61.06 ± 9.89 ^d^
D-M	97.92 ± 18.72 ^a^	56.02 ± 4.53 ^a^	14.65 ± 1.01 ^ab^	0.99 ± 0.06	72.00 ± 1.78 ^a^	103.25 ± 3.82 ^ab^	8.52 ± 0.39 ^b^
D-S	81.12 ± 3.97 ^a^	55.24 ± 1.44 ^a^	14.96 ± 0.79 ^ab^	1.12 ± 0.07	103.17 ± 8.30 ^bc^	120.80 ± 9.78 ^abc^	35.50 ± 7.00 ^ac^
D-C	93.70 ± 7.37 ^a^	56.35 ± 8.82 ^a^	13.07 ± 1.29 ^a^	0.96 ± 0.06	107.20 ± 1.93 ^bc^	107.60 ± 5.13 ^abc^	52.06 ± 3.21 ^cd^
D-F	77.23 ± 3.86 ^a^	49.20 ± 4.17 ^a^	10.74 ± 1.55 ^a^	1.06 ± 0.09	105.80 ± 6.02 ^bc^	100.75 ± 16.42 ^a^	42.47 ± 8.53 ^ac^
D-SC	92.38 ± 6.41 ^a^	47.17 ± 4.39 ^a^	14.13 ± 3.78 ^ab^	1.10 ± 0.14	92.40 ± 4.88 ^b^	105.80 ± 10.39 ^ab^	40.29 ± 2.59 ^ac^
D-SF	81.79 ± 4.57 ^a^	45.37 ± 1.92 ^a^	14.30 ± 2.10 ^ab^	1.05 ± 0.12	102.25 ± 2.95 ^bc^	151.25 ± 18.23 ^c^	39.65 ± 4.94 ^ac^
D-SM	85.36 ± 12.86 ^a^	38.70 ± 4.75 ^a^	14.04 ± 0.69 ^ab^	1.11 ± 0.10	108.60 ± 3.16 ^bc^	133.60 ± 7.13 ^abc^	46.41 ± 4.59 ^cd^
D-CM	82.02 ± 12.92 ^a^	56.34 ± 11.22 ^a^	14.20 ± 1.00 ^ab^	0.87 ± 0.30	110.50 ± 4.72 ^c^	117.00 ± 12.23 ^abc^	49.21 ± 3.48 ^cd^
D-FM	103.74 ± 17.66 ^a^	51.62 ± 5.82 ^a^	14.99 ± 1.46 ^ab^	1.11 ± 0.13	109.33 ± 4.54 ^c^	131.80 ± 14.08 ^abc^	42.71 ± 5.22 ^ac^
D-SCM	102.46 ± 8.86 ^a^	55.02 ± 6.97 ^a^	18.53 ± 1.85 ^bc^	1.04 ± 0.11	113.25 ± 2.50 ^c^	146.50 ± 11.42 ^bc^	46.15 ± 3.04 ^cd^
D-SFM	108.61 ± 18.71 ^a^	108.61 ± 18.71 ^a^	14.68 ± 1.87 ^ab^	1.20 ± 0.23	104.75 ± 7.30 ^bc^	134.50 ± 7.24 ^abc^	39.86 ± 5.97 ^ac^

Aspartate aminotransferase enzyme (AST), alanine aminotransferase (ALT), blood urea nitrogen (BUN), creatinine, total cholesterol (TC), total triglyceride (TG), and low-density lipoprotein (LDL) were analyzed by the One-Way ANOVA followed by the Duncan’s test (n = 6). ^a,b,c,d^ Denotes the different letters indicate significant differences between groups at *p* ≤ 0.05. The data are demonstrated as the mean ± standard error of mean (SEM).

**Table 3:** Effect of SHE, CME, and FCME on anti-inflammatory and antioxidant activity.

Groups	Plasma MDA (µM)	Liver MDA (µM/mg protein)	RBC-GHS (µM)	Liver-GHS (×10^2^ µM/mg protein)	RBC-GPx (µM)	RBC-SOD (µM)
N-DW	1.27 ± 0.15 ^a^	14.75 ± 2.50 ^ab^	85.27 ± 6.76 ^ad^	36.12 ± 11.12 ^a^	705.44 ± 31.24 ^abc^	5.78 ± 0.45 ^a^
D-DW	8.64 ± 0.19 ^d^	46.58 ± 1.64 ^e^	41.45 ± 17.39 ^b^	14.62 ± 4.70 ^bc^	627.18 ± 45.86 ^ab^	1.79 ± 0.16 ^b^
D-M	6.34 ± 0.65 ^c^	21.75 ± 1.23 ^abc^	88.18 ± 7.50 ^d^	25.20 ± 3.32 ^abcd^	646.37 ± 19.63 ^abc^	5.54 ± 0.57 ^a^
D-S	2.46 ± 1.00 ^ab^	40.48 ± 8.22 ^e^	58.95 ± 1.20 ^bc^	27.64 ± 1.66 ^acd^	595.88 ± 26.96 ^a^	5.52 ± 0.32 ^a^
D-C	1.37 ± 0.28 ^a^	33.20 ± 5.75 ^cde^	62.55 ± 2.56 ^c^	30.72 ± 3.71 ^ad^	676.86 ± 55.94 ^abc^	5.34 ± 0.37 ^a^
D-F	1.36 ± 0.09 ^a^	23.97 ± 2.98 ^abc^	65.09 ± 2.30 ^ac^	27.07 ± 2.84 ^abcd^	720.19 ± 44.35 ^bc^	4.63 ± 0.22 ^ae^
D-SC	1.75 ± 0.24 ^a^	39.25 ± 12.68 ^de^	59.18 ± 1.91 ^bc^	23.01 ± 5.06 ^abcd^	673.77 ± 50.36 ^abc^	5.33 ± 0.14 ^a^
D-SF	2.26 ± 0.44 ^a^	26.89 ± 2.18 ^bcd^	62.59 ± 2.30 ^c^	22.60 ± 0.51 ^abcd^	691.46 ± 31.42 ^abc^	2.45 ± 0.38 ^bc^
D-SM	1.56 ± 0.08 ^a^	18.11 ± 0.53 ^ab^	65.09 ± 2.80 ^ac^	17.29 ± 1.67 ^bcd^	759.19 ± 17.11 ^c^	4.12 ± 0.71 ^de^
D-CM	4.00 ± 1.25 ^b^	12.88 ± 1.67 ^ab^	65.41 ± 6.55 ^acd^	13.86 ± 1.81 ^bc^	707.46 ± 32.42 ^abc^	4.02 ± 0.59 ^de^
D-FM	2.71 ± 0.57 ^ab^	11.36 ± 0.74 ^a^	75.27 ± 2.66 ^acd^	14.96 ± 0.53 ^bc^	751.28 ± 30.41 ^c^	2.24 ± 0.19 ^bc^
D-SCM	2.22 ± 0.16 ^a^	13.70 ± 2.03 ^ab^	73.09 ± 10.19 ^acd^	12.72 ± 1.68 ^b^	723.40 ± 8.89 ^bc^	3.05 ± 0.05 ^cd^
D-SFM	1.07 ± 0.13 ^a^	10.82 ± 1.00 ^a^	77.45 ± 2.94 ^acd^	12.55 ± 0.61 ^b^	757.64 ± 25.52 ^c^	2.32 ± 0.24 ^bc^

Plasma malondialdehyde (plasma MDA), liver malondialdehyde (liver MDA), red blood cell blood glutathione peroxidase (RBC-GSH), liver glutathione (Liver-GHS), red blood cell glutathione peroxidase (RBC-GPx), and red blood cell superoxide dismutase (RBC-SOD) were analyzed by the One-Way ANOVA followed by the Duncan’s test (n = 6). ^a,b,c,d,e^ Denotes the different letters indicate significant differences between at *p* ≤ 0.05. The data are demonstrated as the mean ± standard error of mean (SEM).

## Data Availability

The authors declare that the data supporting the findings of this study are available within the article.
